# Clinical and Virological Features of Dengue in Vietnamese Infants

**DOI:** 10.1371/journal.pntd.0000657

**Published:** 2010-04-13

**Authors:** Tran Nguyen Bich Chau, Katherine L. Anders, Le Bich Lien, Nguyen Thanh Hung, Lu Thi Minh Hieu, Nguyen Minh Tuan, Tran Thi Thuy, Le Thi Phuong, Nguyen Thi Hong Tham, Mai Ngoc Lanh, Jeremy J. Farrar, Stephen S. Whitehead, Cameron P. Simmons

**Affiliations:** 1 Oxford University Clinical Research Unit, Hospital for Tropical Diseases, Ho Chi Minh City, Vietnam; 2 Department of Dengue Haemorrhagic Fever, Children's Hospital #1, Ho Chi Minh City, Vietnam; 3 Department of Infectious Diseases, Children's Hospital #2, Ho Chi Minh City, Vietnam; 4 Dong Thap Hospital, Cao Lanh, Vietnam; 5 Laboratory of Infectious Diseases, National Institute of Allergy and Infectious Diseases, National Institutes of Health, Department of Health and Human Services, Bethesda, Maryland, United States of America; Pediatric Dengue Vaccine Initiative, United States of America

## Abstract

**Background:**

Infants account for a small proportion of the overall dengue case burden in endemic countries but can be clinically more difficult to manage. The clinical and laboratory features in infants with dengue have not been extensively characterised.

**Methodology/Principal Findings:**

This prospective, cross-sectional descriptive study of infants hospitalized with dengue was conducted in Vietnam from November 2004 to December 2007. More than two-thirds of 303 infants enrolled on clinical suspicion of dengue had a serologically confirmed dengue virus (DENV) infection. Almost all were primary dengue infections and 80% of the infants developed DHF/DSS. At the time of presentation and during hospitalization, the clinical signs and symptoms in infants with dengue were difficult to distinguish from those with other febrile illnesses, suggesting that in infants early laboratory confirmation could assist appropriate management. Detection of plasma NS1 antigen was found to be a sensitive marker of acute dengue in infants with primary infection, especially in the first few days of illness.

**Conclusions/Significance:**

Collectively, these results provide a systematic description of the clinical features of dengue in infants and highlight the value of NS1 detection for diagnosis.

## Author Summary

Dengue is a major public health problem in tropical and subtropical countries, including Vietnam. Dengue cases occur in children and young adults; however, severe dengue also occurs in infants less than 1 year of age. Prompt recognition of dengue is important for appropriate case management, particularly in infants in whom febrile illness from other causes is common. We describe the clinical picture, virological and immunological characteristics of infants with dengue admitted to three hospitals in southern Vietnam, compared with infants admitted with fever not due to dengue. We show that infants with dengue are difficult to distinguish from those with other febrile illnesses based on signs and symptoms at presentation, and so laboratory tests to confirm dengue virus infection may be useful for diagnosis and management. Conventional diagnostic methods for dengue have low sensitivity early in infection, and we show that an alternative antigen-detection assay that has demonstrated good sensitivity and specificity in older age groups also performs well in infants. This study will help to inform the diagnosis and management of dengue in infants.

## Introduction

Dengue represents a substantial disease burden in many tropical and sub-tropical countries, particularly in children and young adults [1]. Infection with any one of the four dengue virus (DENV) serotypes can lead to a sub-clinical infection, or to clinical disease ranging in severity from a non-specific febrile illness to classical dengue fever, dengue hemorrhagic fever (DHF) and dengue shock syndrome (DSS). Severe dengue (DHF/DSS) is strongly associated with secondary heterotypic dengue virus infections in children and adults [2], [3], but can also occur in primary DENV infection of infants born to dengue-immune mothers [4], [5]. Common to these two epidemiological populations are pre-existing DENV-reactive IgG antibodies, which are thought to be a factor in both serotype-specific immunity to infection as well as the pathogenesis of dengue, through a mechanism of antibody-dependent enhancement of infection [4], [5].

Previous studies have indicated a low incidence of DENV exposure in infants [5], [6], however infants with DHF can be clinically challenging to manage and are at higher risk of mortality than older children [4], [7]. In dengue-endemic areas infants under one year of age comprise between 1–5% of the dengue cases admitted to hospital each year [4], [5], however only a limited number of studies have documented clinical and laboratory findings in infants with dengue [8], [9]. This evidence indicates that the clinical manifestations of dengue in infants may differ from older children and adults, with a greater frequency of low platelet count (<50000 cells/mm^3^), plasma leakage and shock and fewer haemorrhagic manifestations in infants compared with dengue in older children [8], [9]. This paper describes the clinical, hematological and virological characteristics of infants hospitalized with dengue in southern Vietnam. These characteristics were compared to infants hospitalized with other acute febrile illnesses.

## Methods

### Patient recruitment and sample collection

This prospective, descriptive study was conducted at Pediatric Hospitals Number 1 and Number 2, Ho Chi Minh City, and at Dong Thap Hospital, Dong Thap province, Vietnam, from November 2004 to December 2007. Infants under 18 months old with suspected dengue were eligible to be enrolled. Recruitment also took place in the outpatient department of Pediatric Hospital Number 1 from July to December 2005. Written informed consent was obtained from a parent or guardian of each patient. The study was approved by the Scientific and Ethical Committees of the Hospital for Tropical Diseases, Children's Hospital No. 1, Children's Hospital No. 2, and Dong Thap Hospital and Oxford Tropical Research Ethical Committee. Daily venous or capillary blood samples were collected from infants for 4 consecutive days beginning on entry to the study (study day 0), and again 10–14 days after discharge from hospital. Venous blood samples were collected from the mothers of each infant on study day 0 and again 4–8 days later. The extent of hemoconcentration during symptomatic illness was determined by comparing the maximum hematocrit recorded during hospitalization with either the value recorded at follow-up in each patient (47% of cases) or at hospital discharge for those who did not return for follow-up (53% of cases).

### Dengue diagnostics

Dengue antigen capture IgM and IgG ELISA assays used both inactivated DENV and Japanese encephalitis virus (JEV) antigens and monoclonal anti-DENV antibody provided by Venture Technologies (Sarawak, Malaysia) as previously described [10]. Acute DENV infection was defined as DENV-IgM seroconversion (i.e. from negative to positive) between paired specimens, or rising levels of DENV reactive IgM (at least 20% increase in DENV-IgM ELISA Units from 1^st^ to 2^nd^ sample and 2^nd^ sample has ≥20 ELISA Units). World Health Organization (WHO) classification criteria for disease severity [11] were applied to each case after reviewing study notes.

DENV viraemia was measured using an internally-controlled, serotype-specific, real-time RT-PCR assay [12]. Results were expressed as cDNA equivalents per milliliter of plasma. DENV NS1 antigen in plasma was measured using the Platelia NS1 assay (BioRad, Hercules, CA) according to the manufacturers' instructions. The concentration of DENV NS1 protein in test plasma samples was quantified by reference to a standard curve, using serial dilutions of immune-affinity purified recombinant DENV-2 NS1 protein (Hawaii Biotechnology Group, Inc). The limit of detection of this test was 5 ng/ml. DENV viraemia and NS1 antigenaemia were measured in samples collected on four consecutive days from study enrolment.

### Plaque reduction neutralization test (PRNT)

Neutralizing antibody titres against four reference serotypes (DENV-1 PR/94, DENV-2 NGC, DENV-3 Sleman/76, and DENV-4 814699) in maternal plasma samples were determined by a complement-enhanced PRNT assay as described previously [13].

### Statistical analysis

Statistical analyses were performed with SPSS version 14. To compare the distribution of categorical variables between patient groups, a chi-squared test for association was used, with p<.05 considered to be statistically significant. For comparison of continuous variables between groups we used a non-parametric test (Mann Whitney U) or, where there were more than two comparison groups, Kruskal-Wallis one-way analysis of variance. We calculated Spearman's rank correlation coefficients (rho) to examine the strength of association between two continuous variables.

## Results

### Characteristics of the study population

During the study period, we enrolled 293 inpatient infants and young children with suspected dengue and 10 infants treated as outpatients. Seventy-five of these infants have been described previously by our group [13]. The intention of this report is to summarize the main clinical and virological findings in a larger population of infants with dengue. Table 1 shows the demographic characteristics of study participants. Infants' mothers were aged between 19–45 years old (median: 28 yrs), and the infants were aged between 2–18 months old (median: 7 months). The male to female ratio of the infants was 173∶126. The majority of inpatients were enrolled on day 4 or 5 of illness. Sixty-eight percent (204/303) of enrolled infants had serologically confirmed dengue, of which 201 were primary dengue cases and 3 secondary dengue cases. The remaining 99 patients (32%) without serological evidence of dengue were classified as “other febrile illnesses” (OFI). Almost 80% (162/204) of the infants with laboratory confirmed dengue were between 4–10 months old, with the peak at 7–8 months of age.

**Table 1:** Demographic and serological characteristics of study participants.

Features		Dengue (N = 204)	OFI (N = 99)
Median maternal age (range) (yrs)		27 (19–44)	29 (20–45)
Infant age	<4 months	11	5
	4–10 months	163	72
	>10 months	30	22
Sex	Male	115	58
	Female	86	40
Place of enrolment	Ho Chi Minh City	174	66
	Dong Thap	30	33
Hospital ward of enrolment	Inpatient	194	99
	Outpatient	10	0
Median day of illness at enrolment (range)		5 (3–9)	4 (1–7)
Positive DENV reactive IgM at admission	Yes	165	0
	No	35	99
Positive DENV reactive IgG at discharge	Yes	96	0
	No	108	99
Primary/secondary DENV infection	Primary	201	N/A
	Secondary	3	N/A

### Clinical characteristics of infants with laboratory-confirmed dengue

Most of the primary dengue cases were classified as DHF grade II (141/201; 70%), with 17% (35/201) classified as DF and 9% (19/201) classified as DSS (DHF III and IV). Five cases could not be classified by the WHO criteria because of insufficient investigations of vascular leakage. Clinical data was not available for one infant. The five infants in whom disease severity could not be classified were excluded from further analysis.

The clinical features of participants at the time of enrolment are shown in Table 2. Infants with dengue did not present with specific clinical signs compared to patients with other febrile illnesses (OFI). Common features of upper respiratory tract viral infection, including running nose and cough, were observed with similar frequency in both groups, however diarrhea, vomiting and a petechial rash occurred more frequently in dengue patients than in infants with OFI. Fever was observed less frequently in dengue patients; however in some patients enrolled later in their illness, defervescence may have occurred prior to study enrolment.

**Table 2:** Clinical symptoms in dengue and non-dengue patients at the time of study enrolment.

Symptoms	Percent of patients presenting with each symptom				P value*
	Primary dengue (N = 201)		Other febrile illness (N = 99)		
	%	Missing data	%	Missing data	
Fever	83	1	94	0	0.03
Diarrhea	43	1	29	1	0.05
Running nose	37	1	37	0	0.74
Cough	56	3	46	0	0.17
Vomiting	57	4	31	1	<0.01
Jaundice	1	12	0	3	0.34
Petechial Rash	34	3	19	3	0.02
CNS sign	4	11	6	1	0.12

*:Cross tabulation, Chi-square Test.

The hematological findings and clinical manifestations during hospitalization in infants with primary dengue or other febrile illness are shown in Table 3 and Table 4. The median nadir in white blood cell (WBC) count in infants with dengue was significantly lower than that in infants with OFI (5600 vs 6100 cells/mm^3^, P = 0.035) and was primarily due to neutropenia. Liver transaminase levels were significantly higher in dengue cases (Table 3). In comparison with OFI cases, infants with dengue had lower platelet nadirs, greater hemoconcentration, petechiae, bruising, hepatomegaly and clinical evidence of systemic vascular leak (Table 4). The case fatality ratio was approximately 1% among dengue cases. One patient in the OFI group had shock and died of meningitis and pneumonia (Table 4).

**Table 3:** Hematological findings in infants during hospitalization and at follow-up.

	Median (5^th^-95^th^ percentile)			P value (dengue vs OFI)	P value (dengue vs follow-up)
	Dengue	OFI	Follow-up		
WBC^a^ (cells/mm^3^)	5600 (1170–21400)	6100 (1630–21600)	9260 (5590–21600)	0.04	<0.001
Lymphocyte^a^ (%)	67% (39–83)	59% (31–82)	62% (42–74)	0.001	<0.001
Neutrophil^a^ (%)	23% (7–53)	29% (7–64)	27% (16–46)	<0.001	0.01
Monocyte^a^ (%)	12% (2–25)	13% (2–32)	8% (3–13)	0.3	<0.001
AST^b^ (UI/ml)	152 (13–2105)	51 (6–186)	N/A	<0.001	N/A
ALT^b^ (UI/ml)	57 (14–975)	26 (6–89)	N/A	<0.001	N/A
Platelet nadir cells/mm^3^	32000 (13100–92000)	96500 (12000–281000)	N/A	<0.001	N/A

a: minimum values measured in 163/72/72 infants with dengue/with OFI/at follow-up, respectively.

b: maximum values, of 56/62 infants with dengue/OFI, respectively.

**Table 4:** Clinical and haematological characteristics of infants with acute febrile illness.

	Disease groups [number (%)/median (5^th^-95^th^ percentile)]				
Features	DF (N = 35)	DHF (N = 141)	DSS (N = 19)	OFI (N = 99)	P value c
Day of illness at enrolment	4 (3–6)	5 (3–6)	5 (3–6)	4 (2–6)	<0.001
Hepatomegaly	27 (79%)	133 (93%)	19 (100%)	51 (52%)	<0.001
Petechiae	32 (94%)	141 (99%)	19 (100%)	81 (82%)	<0.001
Bleeding a	0	11 (5%)	5 (26%)	7 (7%)	0.10
Bruising	4 (11%)	60 (42%)	10 (53%)	4 (4%)	<0.001
Maximum hemoconcentration (%)	4 [(-8)-16]	23 [8–42]	33 [17–50]	9 [(-8)-27]	<0.001
Clinical leak b	0	51 (36%)	10 (53%)	0	<0.001
Cardiovascular shock	0	0	19 (100%)	2 (2%)	0.01
Blood transfusion	0	1 (0.7%)	6 (32%)	1(1%)	0.16
Death	0	0	2 (10%)	1 (1%)	0.36

*^a^Gum, nose or gastrointestinal bleeding;*

^b^ *Clinical evidence of leak revealed by ultrasound or chest X-ray; (pleural effusions, ascites, gall bladder);*

*^c^differences between Dengue group and OFI group tested by cross tabular Chi square test or Mann-Whitney U.*

### Virological characteristics of infants with primary dengue

RT-PCR was performed on plasma samples collected at enrolment. In plasma samples from the 201 serologically-confirmed primary dengue patients, DENV was detected in 161 (80%). All four serotypes were detected: DENV-1 (66/161, 41%), DENV-2 (72/161, 44%), DENV-3 (22/161, 14%), and DENV-4 (2/161, 1%) (Table 5). The remaining 40/201 (20%) cases were aviraemic at the time of study entry. DENV-1 and DENV-2 were the dominant serotypes in this study population and accounted for 85% (137/161) of viraemic patients.

**Table 5:** Virological characteristics at enrolment in infants with dengue.

	DF (N = 35)	DHF (N = 141)	DSS (N = 19)	Not classifiable (N = 5)	Serotype subtotal (% of total)
Day of illness at enrolment	4 (3–8)	5 (3–9)	5 (3–7)	5 (4–8)	
DENV-1	14	42	8	2	66 (41.5%)
DENV-2	6	59	5	1	71 (43.4%)
DENV-3	4	16	2	0	22 (13.8%)
DENV-4	1	1	0	0	2 (1.3%)
Aviremic	10	23	4	2	
Median (range) viraemia on enrolment (log copies/ml)	4.6 (3.4–8.1)	4.7 (3.4–8.5)	5.1 (3.4–8.5)	4.2 (3.6–4.8)	
Median (range) NS1 antigenaemia on enrolment (log ng/ml)	2.6 (0.8–3.4)	2.5 (0.7–3.9)	2.8 (1.3–3.6)	2.6 (0.8–2.7)	

We compared disease severity in dengue patients infected with different DENV serotypes (Table 5). Although a greater proportion of DENV-2 infections resulted in DHF/DSS (91%) compared to the other serotypes (DENV-1 78%, p = 0.03; DENV-3 82%, p = 0.20; DENV-4 50%, p = 0.05), overall there was no significant association between DENV serotype and severity of disease when DHF and DSS were considered separately (p = 0.22). Four of the 134 infants who developed DHF/DSS were aviraemic at admission. Data on viraemia at enrolment was available for 118/201 infants with primary dengue. We compared viraemia at the time of study enrolment in infants with DF, DHF and DSS and found a non-significant trend towards higher viraemia with more severe disease (p = 0.1; Table 5). However, a limitation of our study was that we did not have samples collected in the first 48–72 hours of illness to be able to measure peak viraemia levels. Instead, we were limited to measuring viraemia at a time when in most patients it was already declining.

**Figure 1:**
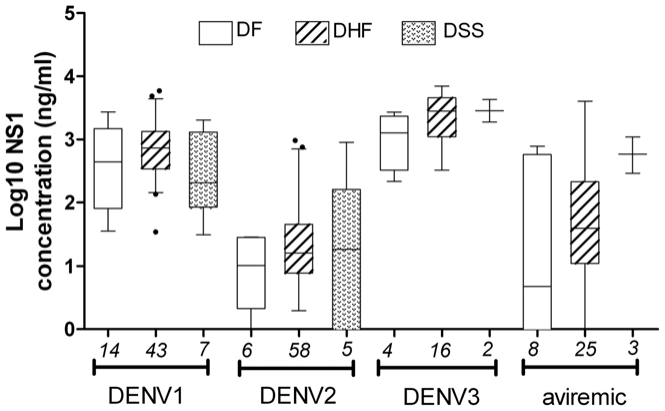
NS1 antigenaemia by disease severity. Shown are the median, interquartile and 95^th^-percentile ranges of log_10_ NS1 concentration at enrolment, distributed by DENV serotypes and disease severity. Day of illness ranged from 3–9 and was comparable among DF, DHF, DSS groups. The number of patients in each group is shown in italics.

The concentration (ng/ml) of DENV NS1 antigen in infant plasma was measured at study enrolment (Table 5). At enrolment, 176/196 (89%) of dengue cases had detectable plasma NS1 levels. The NS1 concentration was significantly higher in DENV-1 and DENV-3 infections than in DENV-2 infections (p<0.001), and did not correlate with viraemia levels, which were similar among DENV serotypes (data not shown). We compared NS1 levels between primary dengue patients of different severities, stratified by DENV serotype (Figure 1). This showed a non-significant trend toward higher NS1 levels with increasing disease severity in those infants infected with DENV-3 or avireamic at admission (Chi-square test: p = 0.2, p = 0.1 respectively), but this trend was not observed in DENV-1 and DENV-2 infected patients.

### The sensitivity of dengue diagnostic tests in acute illness

**Figure 2:**
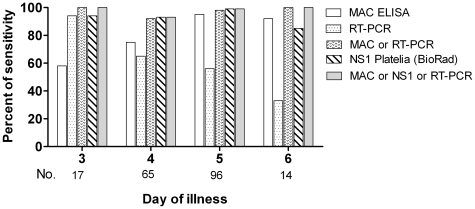
The sensitivity of diagnostic tests in acute DENV infection of infants. The number of patients evaluated on each day of illness is shown below the graph. Bars show the percentage of samples positive by each assay.

The sensitivity of different dengue diagnostic approaches at the time of enrolment were compared to laboratory confirmation by serology on paired samples. NS1 detection was a sensitive approach (>80%) to diagnosing dengue in enrolment plasma samples collected on day 3, 4 or 5 of illness (Figure 2). The sensitivity of the NS1 test was significantly higher than real time RT-PCR in the first 6 days of illness (Chi-square test: p = 0.001). RT-PCR was sensitive early, but sensitivity declined with increasing illness duration. Conversely, IgM detection by MAC ELISA was only moderately sensitive on day 3 and 4, but detection improved with increasing duration of illness.

### DENV neutralizing antibodies in maternal blood and relationship with age of disease onset

Plasma samples from 120 mothers of infants with primary dengue were available for PRNT_50_ assays, after excluding those women with serological evidence of recent DENV infection (n = 33), those with preterm babies (n = 19) and those mothers who did not provide paired blood samples (n = 29). Recent DENV infection was defined as a four-fold change in IgG to recombinant E protein in paired plasma samples or when any maternal plasma sample was IgM positive. Amongst the evaluated maternal plasma samples, 100% had neutralizing antibodies (PRNT_50_ assay) to at least one DENV serotype and 96% of mothers possessed neutralizing antibodies to ≥3 serotypes. The median titer of neutralizing antibody against DENV-4 was much lower than to the other 3 serotypes. The maternal neutralizing antibody titre to DENV1-4 was independent of maternal age (data not shown). Maternal neutralizing antibody titres against the serotype that infected each infant were compared to the infant's age at illness onset. There was only a limited correlation between maternal neutralizing antibody titres and the age at which infants present to hospital with symptomatic primary dengue (Figure S1).

## Discussion

This study describes the clinical and virological features of dengue in Vietnamese infants and is an extension of previous reports from our group [13]. This report highlights the value of NS1 detection in the diagnosis of dengue in infants who otherwise present with non-specific clinical symptoms. Interestingly, we found DENV-2 infections to be associated with significantly lower plasma NS1 levels relative to DENV-1 or DENV-3 infections. Collectively, these data provide new insights into the pathogenesis of severe dengue in an age-group where diagnosis and management can be challenging.

The majority of infants hospitalized with dengue were between four and ten months of age and this is consistent with previous studies at this hospital and elsewhere in SE Asia [4], [5], [8]. Infants with dengue initially presented with non-specific symptoms that included coryza, cough and diarrhea. Subsequently, many infants developed pathognomonic signs of DHF including petechiae, bleeding, bruising and vascular leak. Although the World Health Organization classification criteria were originally developed for older children, 97% of the infants with acute dengue in this study were adequately classified [11]. Consistent with previous studies [8], [9], we demonstrate that DHF/DSS can occur during primary infection of infants. Surprisingly, the patients enrolled in our study had predominantly DHF grade II, with few cases having either DF or DSS. Consequently, we had little statistical power to detect differences in clinical or virological features between mild and severe cases. The limited breadth in our patient population was despite efforts to recruit patients with milder disease presentations by enrolling infants in the outpatients department of Children's Hospital No. 1.

The clinical signs and hematological manifestations in infants hospitalized with dengue were similar to those seen in children and adults [8], [9], [14], [15]. Whole blood count results during hospitalization demonstrated leucopenia and elevated liver transaminases in infants with dengue compared to those with other febrile illnesses, however these differences are unlikely to be sufficient for differential diagnosis at the individual level. Infants with dengue were more likely to have hepatomegaly, petechiae and bruising than infants with other febrile illnesses. Clinical evidence of plasma leak (e.g. ascites) was observed in a minority of infants with DHF/DSS.

The average time to study enrolment since illness onset was 4–5 days. A disadvantage of not enrolling patients into the study earlier in their illness is that it was not possible to identify early risk factors for more severe outcomes e.g. DSS. One of the reasons few infants were enrolled into the study early in their illness relates to the standard of care at the hospitals where the study was conducted. Infants with fevers of just 1–2 days duration are often seen in the outpatient department, but most are treated as outpatients only with daily follow-up. For infants with DHF, only when the characteristic thrombocytopenia and a rising hematocrit are observed, usually on day 3 or 4, are those infants admitted to hospital. Specific dengue diagnostic tests such as PCR or NS1 assays are not routinely available.

By RT-PCR we were able to detect viraemia in 80% of the dengue cases. As supported by previous studies [16], we also found that the proportion of viraemic cases was highest in the first few days of illness. In this study, neither the viraemia nor antigenemia at enrolment correlated with disease severity, although a trend towards higher NS1 concentrations with increasing disease severity was observed in infants infected with DENV-3 or aviraemic at admission. However, our study participants were generally enrolled after 4–5 days of illness and therefore we probably have not measured the peak viraemia or NS1 antigenaemia. A greater proportion of DENV-2 infections resulted in DHF/DSS compared to other serotypes, however the number of DF and DSS cases was small and there was no significant association overall between infecting serotype and disease severity.

NS1 detection may be helpful in diagnosing dengue [17], [18], [19], [20]. In this paper, we illustrated the superior sensitivity of DENV NS1 detection in comparison with two other dengue diagnostic tests (MAC ELISA and real-time RT-PCR), especially in the first few days after disease onset (≤4 days). Early diagnosis could assist clinical management by focusing attention on the clinically important features of dengue (capillary leakage) and minimizing unnecessary use of antibiotics. The sensitivity of NS1 detection was higher than RT-PCR during the first six days of illness, and many infants were still NS1 positive at the time of discharge from hospital. This is consistent with previous descriptions of the sensitivity of NS1 detection in children and adults with primary dengue [17], [20], [21]. One of the reasons NS1 detection might be more sensitive in primary dengue is that the IgM/IgG response does not become measurable until day 6 or later. Previous studies in Vietnamese children demonstrated that a measurable anti-DENV IgG level in the test sample significantly reduced the likelihood of NS1 detection, possibly because of the immune complex formation between anti-NS1 IgG and soluble NS1 in the blood [20]. Viraemia and NS1 antigenaemia appear to correlate closely early in infection, with divergence in the sensitivity of NS1 detection and RT-PCR from day four of illness (Figure 2). This may be due to more rapid degradation of viral RNA compared to NS1 protein. Our results support previous findings [20] of a lower concentration of NS1 antigen in DENV-2 infections, compared with other serotypes, and demonstrate that this is observed also in primary infections in infants as well as in secondary infections, with which DENV-2 is commonly associated. This could be due to the antibodies used in the NS1 ELISA having differential binding to DENV-2 NS1 protein compared with other serotypes and the limitation of using a DENV-2 NS1 protein standard for calibration of the quantitative assay used for all serotypes. This may have resulted in an overestimate of NS1 levels for DENV-1 and DENV-3, however our analysis of the association between NS1 antigenaemia and disease severity was stratified by serotype so is robust to this limitation. Alternatively, DENV-2 infections may result in less soluble NS1 protein due either to a greater tendency for immune complex formation with pre-existing IgG or to differences in viral infection between serotypes.

All of the mothers of infants with primary dengue in this study for whom plasma samples were available possessed DENV-neutralizing antibody, as measured by PRNT_50_. Neutralizing antibody, acquired passively by infants born to dengue-immune mothers, is suggested to provide protective immunity against dengue during the early months of life, but sub-neutralizing levels of maternal antibody are thought to be a factor in the pathogenesis of dengue in infants [22]. We have recently demonstrated that the waning of maternal DENV-neutralizing antibody correlates temporally with the age of peak dengue burden in infants [23]. Building on previous analyses [13] in a small subset of the current study population, we examined the relationship between maternal serotype-specific neutralizing antibody titre and infants' age at infection in individual mother-infant pairs, with the hypothesis that a higher PRNT_50_ titre in the mother may confer a longer period of protection in the infant. A weak positive correlation between maternal anti-DENV-2 PRNT_50_ titre and age at time of disease onset in infants with DENV-2 infections suggested higher maternal neutralizing antibody titres may be associated with a longer window of passively acquired immunity for DENV-2. However, no such correlation was found for DENV-1 or DENV-3. Our findings differ from recently reported observations of a strong positive correlation between maternal DENV-3 PRNT_50_ titre and infant age at the time of symptomatic DENV-3 infection [24]. This difference may be partially attributable to several limitations to the current analysis. Maternal blood at infant admission is an imperfect proxy for infant's maternal antibody titre at birth as, although we excluded any mothers with serological evidence of recent dengue infection, we cannot exclude the possibility of DENV exposure and antibody boosting in the mothers since birth. For reasons of feasibility, and consistent with the work of others [24], we measured PRNT_50_ titres using prototype dengue viruses from each serotype, not viruses isolated from individual infected infants. Although these titres may therefore be an imperfect measure of *in vivo* DENV neutralizing capacity, we believe this to be a reasonable methodology based on the amino acid identity of the DENV E gene between the prototype viruses and dengue viruses isolated in southern Vietnam during the study period (mean 97.8% identity, range 96.3%–98.5% comparing prototype DENV of each serotype to 4 contemporary isolates of that serotype). An important consideration is the fact that the large majority (73%) of dengue cases in our study occurred after 6 months of age. We have reported previously that maternal dengue neutralizing antibody was undetectable (by PRNT_50_) in the majority of a healthy cohort of Vietnamese infants by 6 months of age, regardless of titre at birth [23]. This suggests that after 6 months, infant age at dengue onset may be independent of PRNT_50_ titre at birth if few or no infants have remaining protection from maternal antibody. Further prospective studies of the relationship between passively acquired antibody and the spectrum of dengue infection and disease in infants can contribute to an improved understanding of immune correlates of protection against dengue, and a large prospective birth cohort study is currently underway in southern Vietnam to address these questions.

The data presented in this study highlight the challenges faced by clinicians in distinguishing infants with dengue from those with other febrile illnesses based on clinical and hematological signs alone, and thus identifying infants who may be at risk of developing severe dengue disease. Routine serology is an imperfect tool for the laboratory confirmation of dengue, particularly in the first 1–3 days of illness, and our results indicate that the addition of NS1 detection to the diagnostic algorithm has the potential to improve the early diagnosis of dengue in infants, as well as in older children and adults.

## Supporting Information

Figure S1 Maternal neutralizing antibody and infant age at dengue disease onset. Shown is the serotype-specific 50% plaque-reduction neutralization test (PRNT50) titre in maternal plasma versus the age of individual infants with primary infection with A) DENV-2 (N = 39), B) DENV-1 (N = 38), or C) DENV-3 (N = 14). Each point represents the titre of one mother-infant pair. Only maternal titres to DENV-2 were positively correlated with infant age (Spearman's rho = 0.3 (p = 0.03)).
(0.46 MB TIF)
