# Supplementary figures and images for: Clinical and Virological Features of Dengue in Vietnamese Infants
Source: PLoS Negl Trop Dis. 2010 Apr 13;4(4):e657. doi: 10.1371/journal.pntd.0000657 (PMC2854125; doi:10.1371/journal.pntd.0000657)

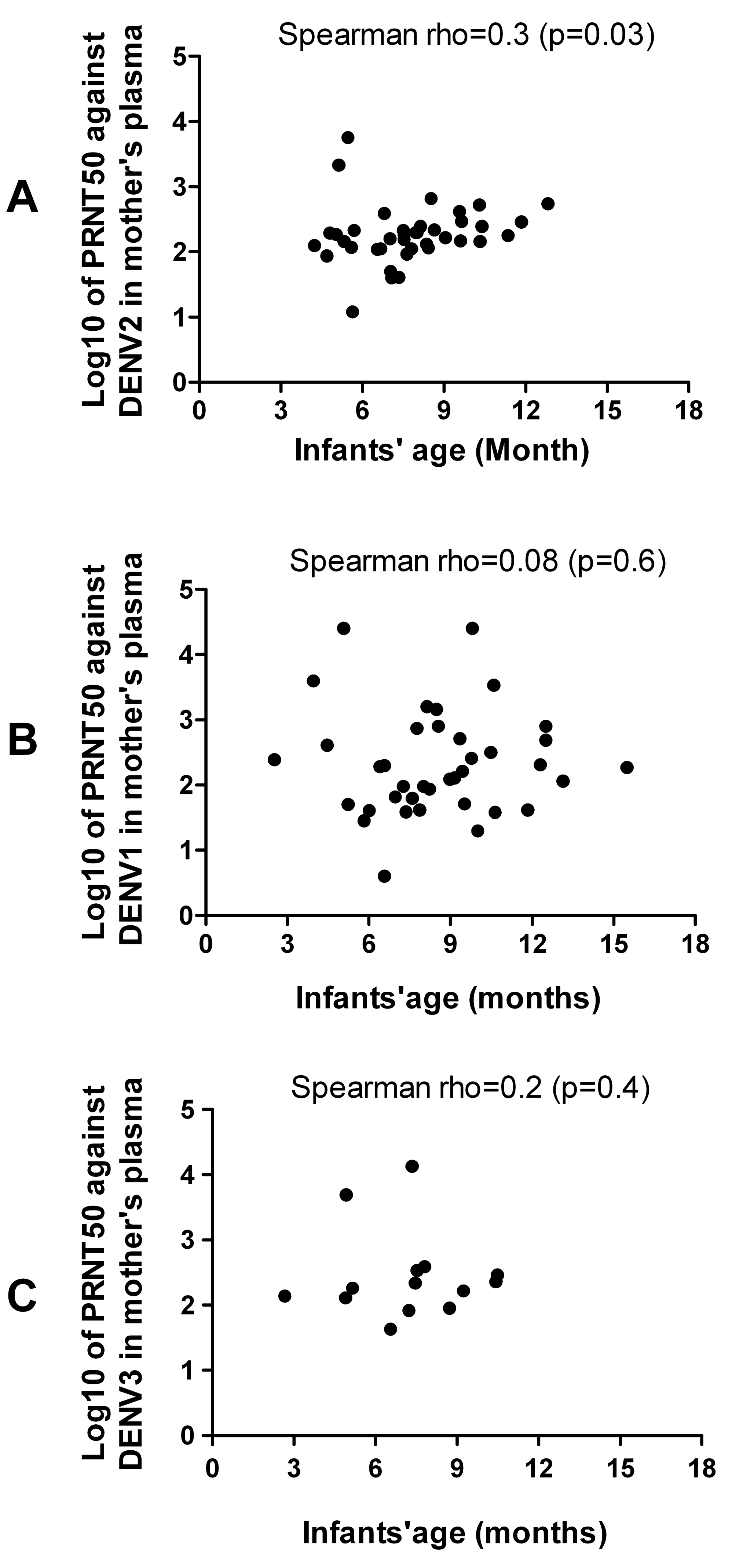

Supplement: Figure S1 — Maternal neutralizing antibody and infant age at dengue disease onset. Shown is the serotype-specific 50% plaque-reduction neutralization test (PRNT50) titre in maternal plasma versus the age of individual infants with primary infection with A) DENV-2 (N = 39), B) DENV-1 (N = 38), or C) DENV-3 (N = 14). Each point represents the titre of one mother-infant pair. Only maternal titres to DENV-2 were positively correlated with infant age (Spearman's rho = 0.3 (p = 0.03)). (0.46 MB TIF) [file pntd.0000657.s001.tif]
